# The conundrum of acute chest pain in general practice: a nationwide survey in The Netherlands

**DOI:** 10.3399/bjgpopen18X101619

**Published:** 2018-11-28

**Authors:** Ralf Harskamp, Petra van Peet, Jettie Bont, Suzanne Ligthart, Wim Lucassen, Henk van Weert

**Affiliations:** 1 Postdoctoral Researcher and GP Registrar, Department of General Practice, Academic Medical Center, Amsterdam, The Netherlands; 2 GP and Associate Professor, Department of Public Health and Primary Care, Leiden University Medical Center, Leiden, The Netherlands; 3 GP and Head of GP-specialty Training Programme, Department of General Practice, Academic Medical Center, Amsterdam, The Netherlands; 4 GP and Researcher, General Practice Oosterhout, Nijmegen, The Netherlands; 5 GP and Senior Researcher, Department of General Practice, Academic Medical Center, Amsterdam, The Netherlands; 6 GP and Head of the Department of General Practice, Academic Medical Center of Amsterdam, Amsterdam, The Netherlands

**Keywords:** Chest pain, general practice, acute coronary syndrome, survey, point-of-care test, clinical decision aid

## Abstract

**Background:**

GPs are frequently confronted with patients with acute onset chest pain. Although usually benign, approximately 5% is due to acute coronary syndrome (ACS). Unfortunately, ACS is not always recognised, leading to a missed diagnosis in 2–5% of presentations.

**Aim:**

The authors set out to study the level of risk GPs are willing to accept with regards to missing an ACS diagnosis, and the receptiveness of implementing new clinical decision aids.

**Design & setting:**

This study involved an online survey among GPs in the Netherlands.

**Method:**

A concept survey was constructed, which was tested among a panel of 24 GPs. The survey was then modified to achieve content validity. This survey was electronically distributed among 1000 GPs.

**Results:**

A total of 313 (31.3%) GPs completed the survey. Of those surveyed, the median age was 50 years (interquartile range 41–57), 53.0% were female, and 6.4% were specialist GPs ('kaderarts') in cardiology or acute care. GPs estimated the missed ACS rate to be <5.0% in clinical practice, most often estimating a chance of 1.0–2.5% (35.2%) or 0.5–1.0% (29.7%). For atypical case presentations, 70% of GPs would accept a 0.1–1.0% missed diagnosis rate, while keeping the referral threshold to a maximum of 50 unnecessary referrals for each ACS case (75% of responders). GPs would welcome additional decision aids, with 79.2% favouring a clinical decision aid, 77.1% favouring troponin point-of-care (POC) testing, and 85.5% favoring a combination of a clinical decision aid and a troponin POC test.

**Conclusion:**

GPs perceive that they miss more ACS cases than they feel comfortable with, which is reflected in a defensive referral strategy. The vast majority of GPs would welcome the use of clinical decision aids and/or cardiac biomarker POC testing for ruling out ACS, if accompanied by more certainty than based on clinical judgment alone.

## How this fits in

GPs are very much aware that clinical judgment is fallible in the setting of acute onset chest pain. This survey evaluated what balance GPs are willing to accept when it comes to the number of referred patients versus the risk of missing ACS. The survey also indicates that GPs would be highly receptive of implementing reliable clinical decision rules and POC tests into their practice.

## Introduction

Chest pain is a common symptom in general practice, accounting for 0.7–2.7% of all consultations.^1–4^ Although the underlying cause is usually benign, potentially life-threatening conditions, such as ACS, may be present in 1.5–10% of all chest pain cases.^1,2,4^ The challenge for GPs is to make an accurate diagnostic assessment while being aware of variations in clinical presentation, having access to limited resources, having time restraints, and operating in a changing society with increased concern of medicolegal consequences. GPs act by overestimating the risk of ACS at initial assessment and using a low referral threshold.^2^ However, GPs cannot refer each and every patient with chest pain and, as such, there is a need to come to terms with what ratio GPs are willing to accept when it comes to the number of referred patients versus the risk of missing ACS. Consensus among GPs of an acceptable risk of missing ACS subsequently influences the sensitivity level required for new diagnostic strategies for ruling out ACS. The authors, therefore, conducted a survey in which they evaluated the level of uncertainty and risk of missing a diagnosis of ACS that GPs are willing to accept with regards to acute chest pain. In addition, the receptiveness of GPs to implementing promising clinical decision aids to help avoid missing ACS in the setting of chest pain was assessed.

## Method

An electronic survey instrument was created regarding the diagnostic dilemma of acute chest pain in general practice. The survey questions arose in multiple brainstorm sessions with the research group and the research team subsequently discussed these with cardiologists. The survey consisted of four parts: (1) assessing knowledge of current percentages of missed ACS diagnosis in clinical practice; (2) identifying what rate of missing the diagnosis ACS in general practice is considered acceptable; (3) assessing clinical triggers that influence the referral threshold; and (4) the receptiveness to implementing clinical decision aids in acute chest pain. To achieve face validity, user feedback was obtained through a pilot survey. The pilot survey was circulated among 24 GP (registrar)s. The pilot survey users were asked whether the introductory statement clearly outlined the purpose of the survey and whether it was motivational, and they provided an estimate of the time required to complete it. For each question the authors asked whether the interviewee judged the question to be relevant for GPs, to be presented in such a way that a reader could easily comprehend the question, and whether the survey included any double-barreled questions. Responders were also allowed to propose additional questions. The survey was then modified to achieve content validity and a final version with 13 questions was formatted. An original or English-translated version of the survey form, the original dataset, and statistical codes are available from the authors on request.

The survey was conducted using a random sample of 1000 email addresses retrieved from a digital database of GPs in the Netherlands. The survey was conducted using online survey development cloud-based software (SurveyMonkey). An invitation email was sent out on 5 February 2018, a reminder email was sent on 9 February 2018 and a closing email on 14 February 2018. The survey was closed on day 14 (19 February 2018).

The collected survey data were downloaded and stored as a data document on one of the secure local servers at the Academic Medical Center in Amsterdam, The Netherlands. Descriptive data analyses were subsequently performed using SPSS statistical software package (version 24.0).

### Patient and public involvement

Patients, patient advocates, and/or advisers were neither involved in the development nor in the conduct of this survey. This input was deemed irrelevant given that the focus of the study was specifically targeting the attitudes and perception of GPs in relation to a diagnostic dilemma.

## Results

A total of 392 of the 1000 invitations were answered. Of the responders, 62 opted out (declined participation) and 330 started the online survey. Of the 330 responders, 313 (94.8%) completed all questions. The average survey completion time was 4 minutes.

### Characteristics of responders

An overview of the characteristics is listed in Table 1. In summary, the median age of the responders was 50 years and 53.0% were female. Experience, expressed as years in practice, ranged from 0–42 years, with a median of 17 years. Overall, these characteristics corresponded well with national GP registration data.^5^ Twenty of the responders (6.4%) were GPs specialising in cardiovascular or acute care. The majority of responders practised in a (small) town (≤100 000 inhabitants). The geographic distribution of the responders corresponded well with the distribution of the Dutch population, with the highest response density in the western and central parts of the country.

**Table 1.** Responders’ characteristics

Characteristic	*n* (%)
**Median age, years (IQR)**	50 (41–57)
**Female**	175 (53.0)
**Median years in practice (IQR)**	17 (8.5–24)
**Profession**	
GP	260 (83.1)
GP specialised in cardiovascular care	17 (5.4)
GP specialised in acute care	3 (1.0)
GP with other specialty	3 (1.0)
Retired GP (<5 years)	12 (3.8)
GP trainee	18 (5.8)
**Nearest hospital**	
<5 km	127 (40.6)
5–10 km	80 (25.6)
10–20 km	81 (25.9)
20–30 km	21 (6.7)
>30 km	4 (1.3)
**Practice location**	
Rural	57 (18.2)
Small town (<50 000 inhabitants)	125 (39.9)
Town (50 000–100 000 inhabitants)	68 (21.7)
City (>100 000 inhabitants)	55 (17.6)
Other	8 (2.6)

IQR = interquartile range.

### Estimates on risk of missed diagnosis of ACS

**Figure 1.**
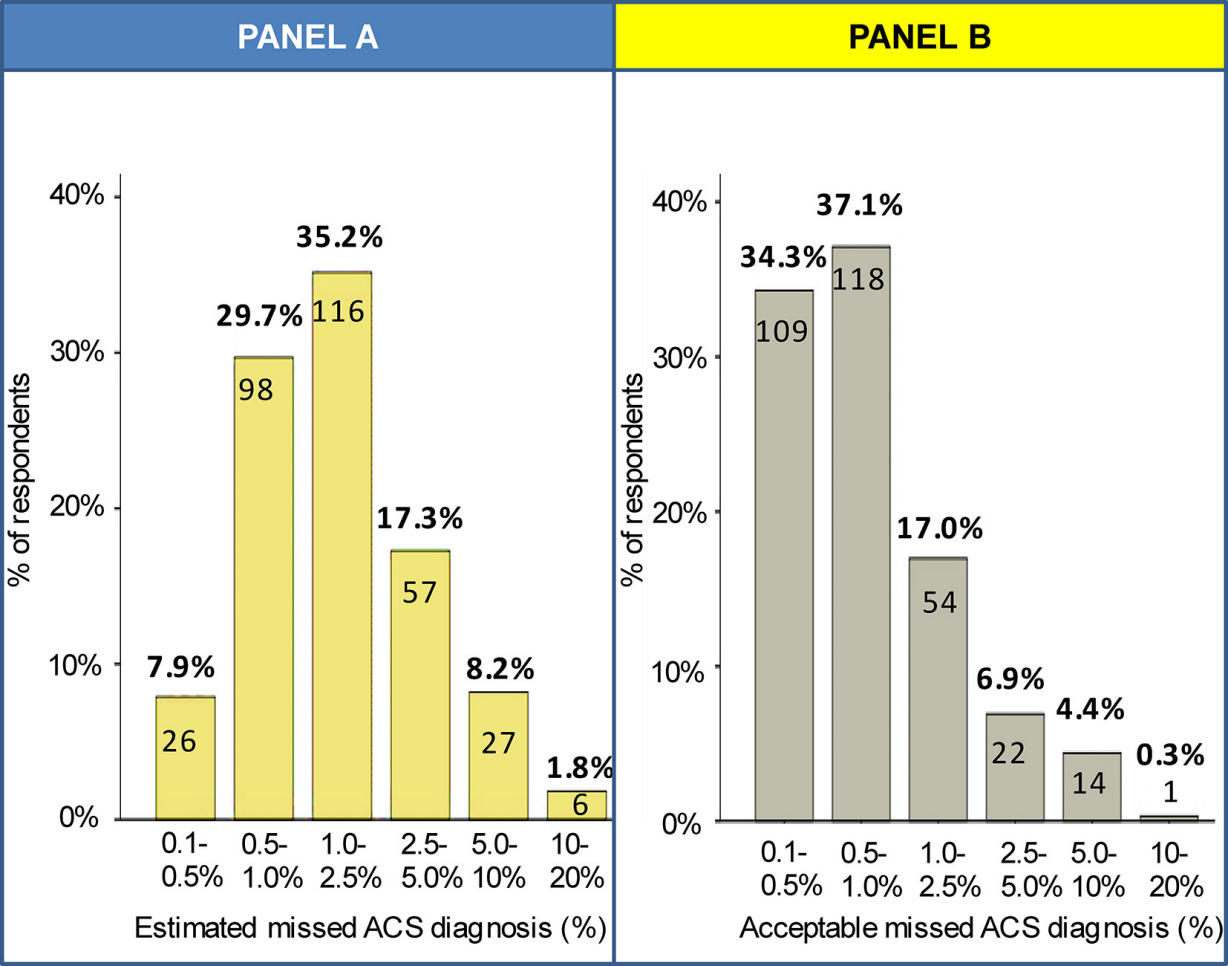
Panel A (left) displays the estimated percentage of failed-to-recognise ACS. Panel B (right) displays the acceptable failed-to-recognise ACS risk for atypical presentations. ACS = acute coronary syndrome.

As shown in Figure 1A, most GPs estimated that the percentage of missed ACS would be between 0.5% and 5.0% (82.2%). The most chosen category was 1.0–2.5% ACS missed diagnosis rate (35.2%). Responders were more favourable about the odds their specialist colleagues at the (cardiac) emergency room would have for missed diagnosis of ACS and other major adverse cardiac events (not shown in figure). Over half of GPs (54.8%) estimated a risk of 0.1–0.5% of missing a major adverse cardiac event for patients referred for acute chest pain.

GPs were split on whether the chance of missing the diagnosis ACS would differ between patients they evaluate in their own practice versus during shifts at out-of-hours GP clinics. In the out-of-hours clinics, patients are triaged centrally and an ambulance is frequently sent immediately to the patient without consulting the GP. Sometimes additional testing possibilities (that is, electrocardiogram [ECG]) are available at the clinic. Of the responders, 36.8% and 41.0% believed the risk of missing ACS was respectively higher or lower at out-of-office-hours GP clinics compared with their own practice, while 22.2% stated no difference in risk of misdiagnosis. Out of the responders who perceived a difference in chance of missing ACS (*n* = 256), 162 responders also provided a reason for this perceived difference. These reasons are outlined in Table 2.

**Table 2.** Reasons for perceived difference in risk of missed ACS between out-of-hours GP service and day-time practice (*n* = 162)

Reason	Higher risk of missed ACS in out-of-hours GP setting, (*n* = 70), *n* (%)	Lower risk of missed ACS in out-of-hours GP setting, (*n* = 92), *n* (%)
Unfamiliarity with patient, past medical history, context	49 (70.0)	18 (19.6)
Defensive stance and triage policy at out-of-office facility	3 (4.3)	54 (58.7)
Less experienced GPs at out-of-office facility	3 (4.3)	0 (0.0)
Higher a priori chance of ACS	9 (12.9)	7 (7.6)
Easy access to emergency department or hospital	1 (1.4)	9 (9.8)
Time pressure at out-of-office facility	2 (2.9)	0 (0.0)
Availability of electrocardiogram	3 (4.3)	1 (1.1)
No opportunity for follow-up	0 (0.0)	3 (3.3)

ACS = acute coronary syndrome.

### The balance of risk for ACS and unnecessary referrals

**Figure 2.**
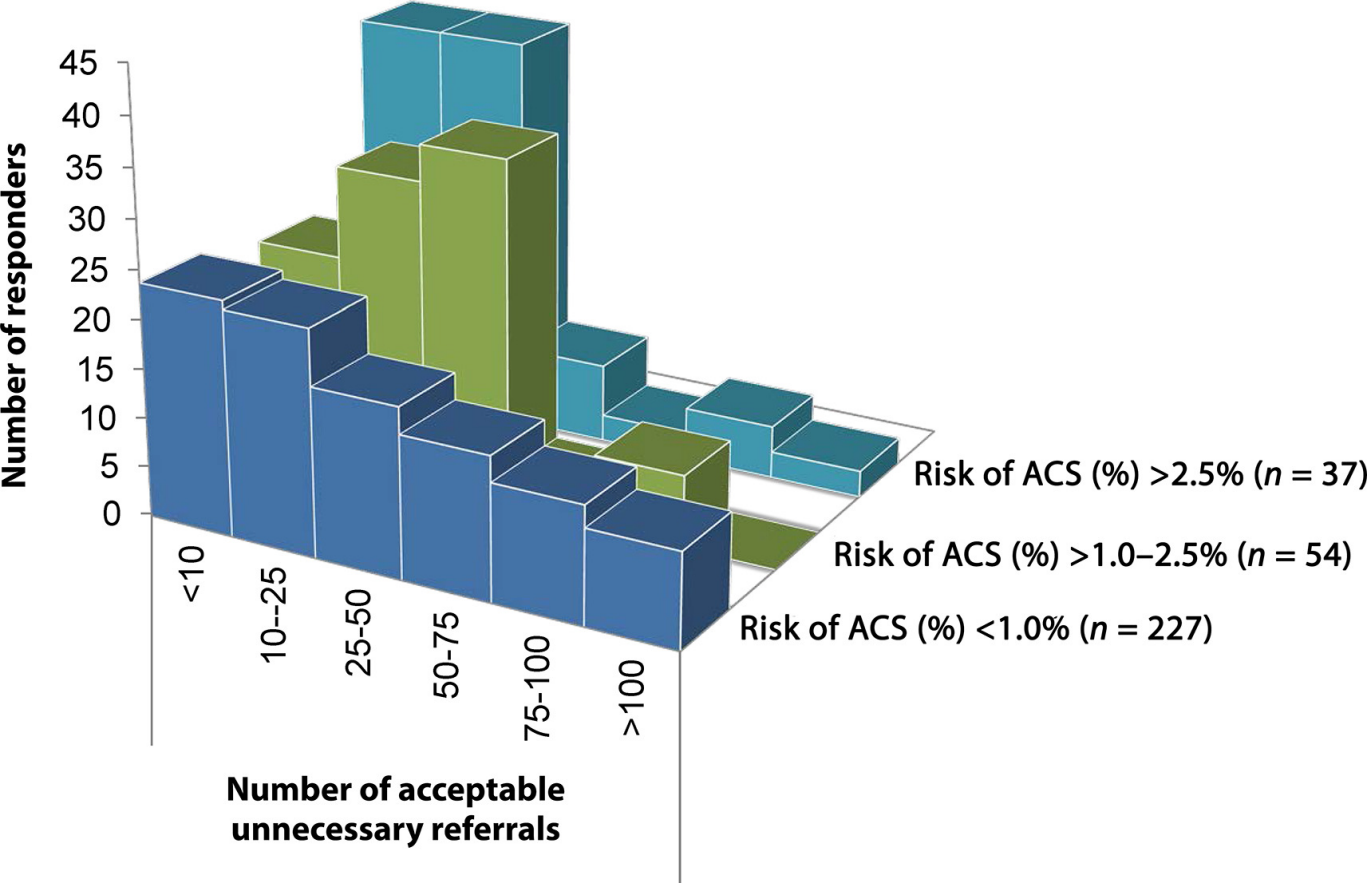
Number of referrals stratified by acceptable risk of failed-to-recognise ACS among atypical presentations. ACS = acute coronary syndrome.

GPs were subsequently asked what risk they would accept for missing a diagnosis of ACS in a patient in whom typical symptoms associated with ACS were lacking. The distribution is displayed in Figure 1B. Overall, GPs would accept a risk of (far) less than 2.5%, in which the risk categories of 0.1–0.5% (34.3%) and 0.5–1.0% (37.1%) were equally common as maximum acceptable risk. Less than 5% of responders would accept the risk exceeding 5.0%. When asked, how many (in hindsight) unnecessary referrals they would view as acceptable per ACS case, the answer varied considerably. The most common answer was a ratio of 10–25 referrals (26.4%) for each ACS case, followed by 5–10 (21.1%) and 25–50 referrals (19.2%), respectively. Figure 2 illustrates that those responders who accepted a great ACS risk also tended to be more reluctant in referring patients.

### Characteristics that influence referral decisions

**Figure 3.**
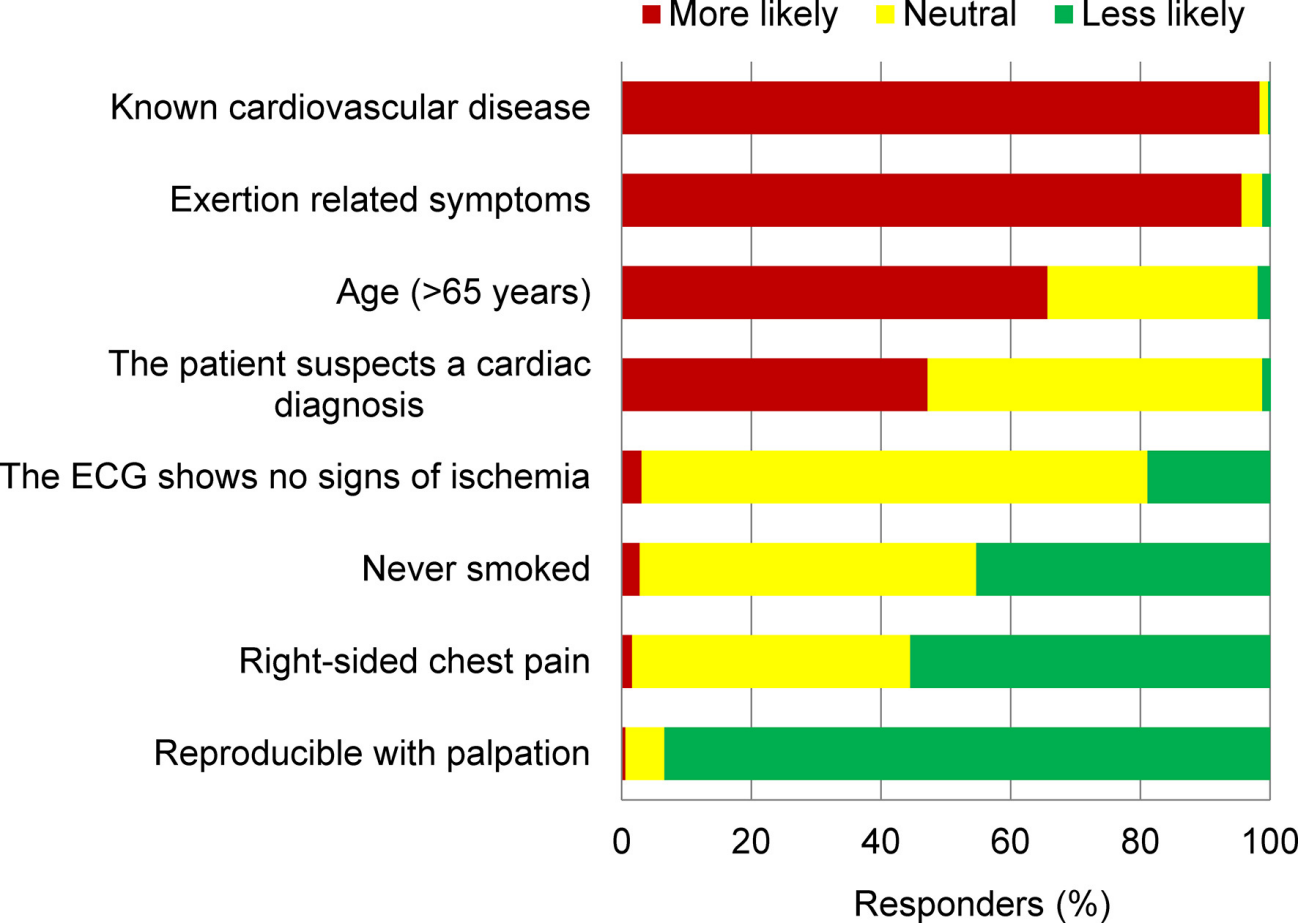
Characteristics that influenced the GP’s likelihood for referral. ECG = electrocardiogram.

The authors also surveyed a number of patient characteristics and symptoms that they considered to be of influence when making referral decisions. Figure 3 displays how the responding GPs felt these characteristics and symptoms would influence their decision making. A past medical history of cardiovascular disease (98.4%) and exertion-related symptoms (95.6%) strongly influenced the likelihood to refer for urgent cardiac evaluation. Older age (>65 years, 65.7%) and patient suspecting a cardiac diagnosis (47.2%) also increased the likelihood for referral. The finding that chest pain could be reproduced with palpitation would strongly influence the decision not to refer (93.4%). Responders were also less inclined to refer in the case of right-sided chest pain (55.5%) and when the patient had no history of smoking (45.3%). An ECG without signs of ischaemia did not influence referral decision making in most responders (78.0%).

### Attitudes towards clinical decision aids and POC testing

**Figure 4.**
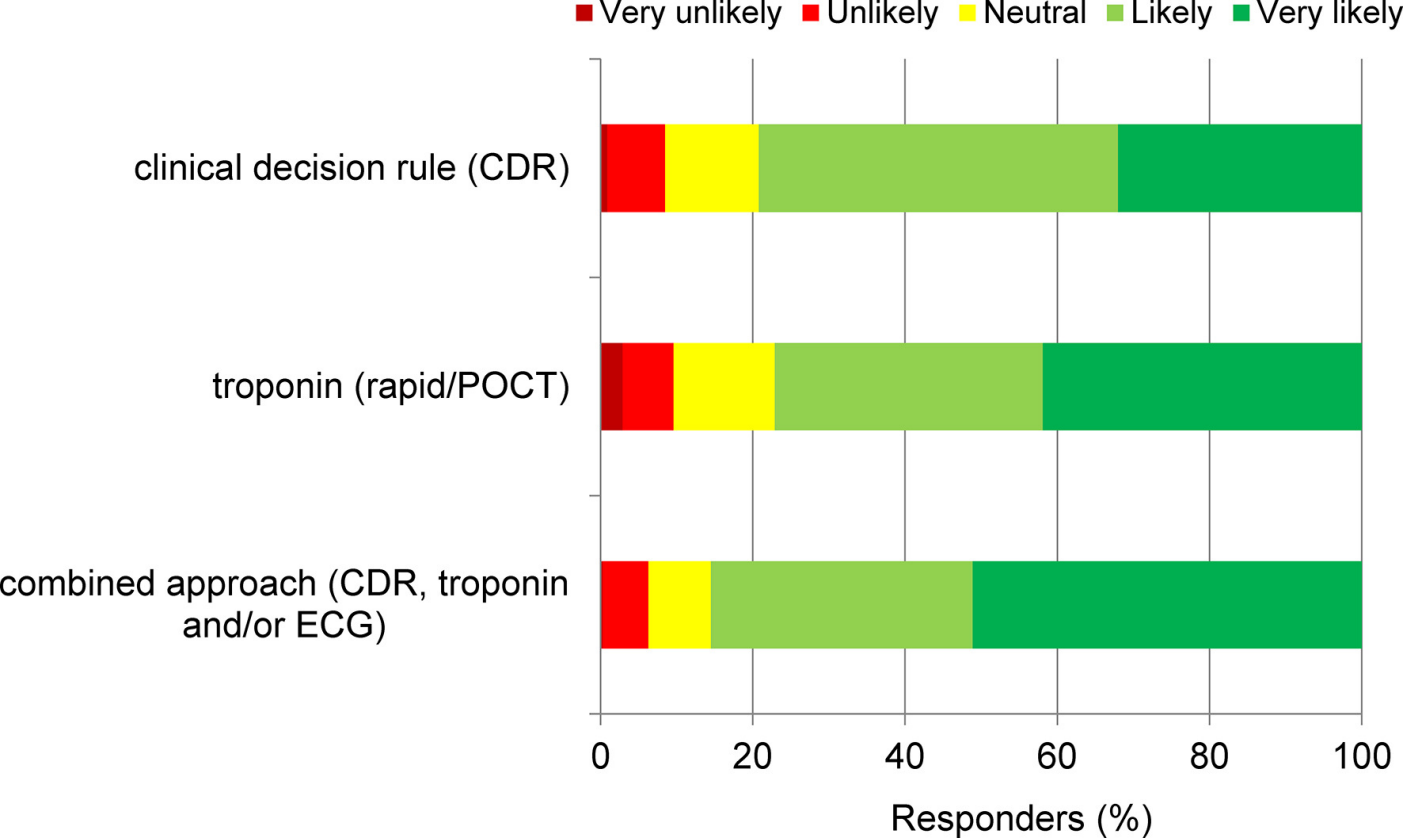
Attitude towards implementing clinical decision aids and POCTs. ECG = electrocardiogram. POCT = point of care test.

*Figure 4* displays GPs’ attitude towards implementing a clinical decision aid, a cardiac biomarker POC test, and/or a combined approach into their daily practice. Responders were favourable towards the use of a clinical decision rule (47.2% likely, 32.0% highly likely to use) and the use of a troponin POC test (35.2% likely, 41.9% highly likely to use). The combined use of clinical decision rule and a troponin POC test and/or ECG was most favoured (34.4% likely, 51.1% highly likely to use). The responders commented (*n* = 117) that these tools should be viewed as supplementary to clinical judgment and not as a substitute. Concerns expressed were the risk of false-negative results for ECG and troponin in early onset or chest pain of short duration; time constraints; and feelings of incompetence (in reading ECGs). Some GPs reported that they consider it the cardiologist’s job to rule out ACS, also when carrying a low clinical suspicion. Interestingly, 11 responders (out of 117 responders to comments box) stated that they already use a combination of clinical judgment with a clinical decision rule, ECG, and/or troponin test.

## Discussion

### Summary

To the authors' knowledge, this survey presents the first systematic assessment to gain insight into the perception and attitude towards diagnostic risk assessment of acute chest pain in a large sample of GPs. It provides useful data on the balancing act of GPs when weighing the risk of missing a diagnosis of ACS versus unnecessary referral. Most GPs would accept a missed diagnosis rate of 0.1–1.0% for ACS, which is substantially lower than the estimated current missed diagnosis rate of 0.5–5.0%. Also, four out of five GPs would welcome new tools, such as decision aids and/or POC tests, to assist them in clinical decision making.

### Strengths and limitations

To maximise the response rate, a 5-minute survey was conducted using a multiplatform accessible electronic format. This resulted in a response rate of >30%, which is considered a quite reasonable result in GP practice. Also the baseline characteristics and geographical distribution suggest a representative sample of GPs in the Netherlands. However, selection bias remains a matter of concern, as more outspoken (owing to personal experience) and/or otherwise motivated GPs were more likely to respond than those who are less outspoken in their opinions. Moreover, while the online survey had the potential to increase ease of response within the target population, some GPs may have not participated because of their discomfort with online interactions. While the authors carefully designed and conducted this survey and, as a result, obtained valuable data that offered insight into attitudes and opinions about the dilemma of chest pain, it remains subject to interpretation and does not provide a full picture.

### Comparison with existing literature

This survey demonstrates that GPs are somewhat optimistic when it comes to the ACS-misdiagnosis rates in general practice. In the literature, 0.7–2% cases of chest pain during regular surgery hours were initially suspected of a non-urgent diagnosis while in fact ACS was present.^2,6^ When out-of-hours GP clinics are also included, the missed diagnosis rate appears to be higher. Data from a Dutch cohort (*n* = 298) found a missed ACS diagnosis rate of 8.2%.^7^ This percentage was also found in a study among 25 Flemish GPs in the early 1990s.^8^

The present survey found that GPs were also optimistic when it came to the rate of missing major adverse cardiac events at the emergency department, which GPs assessed to be <0.5%, which in most cases when using modern clinical decision rules (of which the HEART and T-MACS scores are most popular in Europe) would come down to 0.7–1.7%.^9^ A percentage of <1% is considered an acceptable miss rate by most emergency physicians.^10–12^ Similar to emergency physicians, GPs should also reach consensus on what miss rate for ACS is acceptable.

This survey shows that GPs do not seem to accept the status quo and would rather aim for a 0.1–1.0% missed diagnosis rate for ACS, which approaches that of the emergency department. GPs also strive, by and large, to keep the number of unnecessary referrals to a maximum of 25–50. Based on the GP’s clinical judgment alone, achieving these targets would be a utopia. One could only reach a <1.0% missed diagnosis rate when virtually every patient is referred for secondary evaluation; a costly and undesirable scenario. On the other hand, when accepting a maximum of 25–50 unnecessary referrals, one has to accept a missed diagnosis rate that is in the 1–5% range, depending on the population. Could the use of clinical decision rules and/or POC biomarker and/or ECG testing perhaps close the gap between the actual and targeted missed diagnosis rate? An approach that would involve more advanced diagnostic tools is certainly welcomed by the majority of responders in this survey.

In the past decade, a number of efforts have been made in developing clinical decision aids in general practice settings. Of those, the Marburg Heart Score^13^ and INTERCHEST score^14^ hold most promise, with negative predictive values of 97–98% in general practice populations. However, the caveat is that patients with chest pain of acute onset were underrepresented in the derivation and validation cohorts, and the end diagnosis was not determined with gold standard diagnostic measures. Interestingly, the GPs in this survey already apply a number of the variables of these clinical decision rules in their clinical judgment (namely, older age, known cardiovascular disease, exertion-related symptoms, patient suspecting a cardiac diagnosis, and palpitation reproducible symptoms).

This survey indicates that almost one in five responders would be less likely to refer a patient when the signs of ischaemia are absent on the ECG. There is some validity to that judgment; although, a word of caution is necessary as in the early stages of ACS, the ECG may appear normal. Prior research has shown a sensitivity and specificity of 76% and 88% respectively for out-of-hospital ECG for the diagnosis ACS.^15^ When extrapolating these results to a GP setting with a low a priori chance of ACS, the negative predictive value would still not exceed 97%. When examining cardiac biomarkers, most data are from the emergency or hospital setting, where the use of high-sensitivity cardiac troponin assays have been shown to have a negative predictive value of 98–99%.^16,17^ For the ambulatory setting, POC tests have been developed for conventional (non-high-sensitivity) troponin and the early marker heart-fatty acid binding protein (H-FABP). Studies in general practice found a negative predictive value for H-FABP^18^ and for troponin T, 94–96% for ACS^19–21^ and 99.0–99.7% for myocardial infarction, respectively.

### Implications for research and practice

Future studies are warranted to investigate whether the combined use of a clinical prediction rule (such as Marburg Heart Score) and a troponin and/or H-FABP POC test would lead to a more effective evaluation and safer exclusion of ACS. An exploratory study by Willemsen *et al* found that a combined decision aid based on H-FABP, ECG, and selected symptoms resulted in a negative and positive predictive value of 97.2% and 17.7%, respectively for ACS.^22^ However, the question remains whether GPs will be able to reach this target even with these diagnostic aids, or whether they should settle for a more realistic goal. Kline *et al* ^23^ suggest that a 2% miss rate for ACS should be considered acceptable based on the testing threshold at which the risk of harm from further testing equals or exceeds the chance of benefit from confirming ACS. Future research may reveal whether GPs have to settle for this target or whether they can beat the odds. However, science will not provide the answers on what ratio (number of all referred patients versus number of not referred patients with ACS) should be considered acceptable. To answer this question there is a need for a much broader audience, which would allow discussion of these matters not only within but also outside medical communities. Setting and collectively embracing a realistic goal could perhaps also take off some of the medicolegal pressure that doctors feel when evaluating a patient with chest pain.

In conclusion, physicians are well aware of the current missing rate of ACS when evaluating patients with chest pain of recent onset. GPs also perceive that they miss more ACS cases than they would feel comfortable with. Most GPs would strive for a 1% miss rate, but not at the expense of many unnecessary referrals. This study may serve as a first step towards consensus on what level of uncertainty (and missed diagnosis) GPs are willing to accept with regards to acute chest pain in general practice. Finally, the vast majority of GPs would embrace the use of clinical prediction rules and/or cardiac biomarker POC testing for ruling out ACS with more certainty than based on clinical judgment alone.

## References

[1] Haasenritter J, Biroga T, Keunecke C (2015). Causes of chest pain in primary care — a systematic review and meta-analysis. Croat Med J.

[2] Hoorweg BB, Willemsen RT, Cleef LE (2017). Frequency of chest pain in primary care, diagnostic tests performed and final diagnoses. Heart.

[3] Nilsson S, Scheike M, Engblom D (2003). Chest pain and ischaemic heart disease in primary care. Br J Gen Pract.

[4] Verdon F, Herzig L, Burnand B, GMIRG (2008). Chest pain in daily practice: occurrence, causes and management. Swiss Med Wkly.

[5] van Hassel DTP, Kasteleijn A, Kenens RJ (2016). *Cijfers uit de registratie van huisartsen* [Data from general practice records]. https://www.nivel.nl/sites/default/files/cijfers-uit-de-registratie-van-huisartsen-peiling-jan-2015.pdf.

[6] Bösner S, Haasenritter J, Abu Hani M (2010). Accuracy of general practitioners' assessment of chest pain patients for coronary heart disease in primary care: cross-sectional study with follow-up. Croat Med J.

[7] Bruins Slot MH, Rutten FH, van der Heijden GJ (2011). Diagnosing acute coronary syndrome in primary care: comparison of the physicians' risk estimation and a clinical decision rule. Fam Pract.

[8] Buntinx F, Truyen J, Embrechts P (1991). Chest pain: an evaluation of the initial diagnosis made by 25 Flemish general practitioners. Fam Pract.

[9] Poldervaart JM, Langedijk M, Backus BE (2017). Comparison of the GRACE, HEART and TIMI score to predict major adverse cardiac events in chest pain patients at the emergency department. Int J Cardiol.

[10] Than M, Herbert M, Flaws D (2013). What is an acceptable risk of major adverse cardiac event in chest pain patients soon after discharge from the Emergency Department? A clinical survey. Int J of Cardiol.

[11] Backus BE, Six AJ, Kelder JC (2013). A prospective validation of the HEART score for chest pain patients at the emergency department. Int J Cardiol.

[12] Body R, Carlton E, Sperrin M (2017). Troponin-only Manchester Acute Coronary Syndromes (T-MACS) decision aid: single biomarker re-derivation and external validation in three cohorts. Emerg Med J.

[13] Haasenritter J, Donner-Banzhoff N, Bösner S (2015). Chest pain for coronary heart disease in general practice: clinical judgement and a clinical decision rule. Br J Gen Pract.

[14] Aerts M, Minalu G, Bösner S, International Working Group on Chest Pain in Primary Care (INTERCHEST) (2017). Pooled individual patient data from five countries were used to derive a clinical prediction rule for coronary artery disease in primary care. J Clin Epidemiol.

[15] Ioannidis JP, Salem D, Chew PW (2001). Accuracy and clinical effect of out-of-hospital electrocardiography in the diagnosis of acute cardiac ischemia: a meta-analysis. Ann Emerg Med.

[16] Body R, Burrows G, Carley S (2015). High-sensitivity cardiac troponin t concentrations below the limit of detection to exclude acute myocardial infarction: a prospective evaluation. Clin Chem.

[17] Mueller C (2014). Biomarkers and acute coronary syndromes: an update. Eur Heart J.

[18] Bruins Slot MH, Rutten FH, van der Heijden GJ (2013). Diagnostic value of a heart-type fatty acid-binding protein (H-FABP) bedside test in suspected acute coronary syndrome in primary care. Int J Cardiol.

[19] Nilsson S, Andersson PO, Borgquist L (2013). Point-of-care troponin T testing in the management of patients with chest pain in Swedish primary care. Int J Family Med.

[20] Planer D, Leibowitz D, Paltiel O (2006). The diagnostic value of troponin T testing in the community setting. Interl J Cardiol.

[21] Tomonaga Y, Gutzwiller F, Lüscher TF (2011). Diagnostic accuracy of point-of-care testing for acute coronary syndromes, heart failure and thromboembolic events in primary care: a cluster-randomised controlled trial. BMC Fam Pract.

[22] Willemsen RT, Buntinx F, Winkens B, ‘RAPIDA’-study team (2014). The value of signs, symptoms and plasma heart-type fatty acid-binding protein (H-FABP) in evaluating patients presenting with symptoms possibly matching acute coronary syndrome: background and methods of a diagnostic study in primary care. BMC Fam Pract.

[23] Kline JA, Johnson CL, Pollack CV (2005). Pretest probability assessment derived from attribute matching. BMC Med Inform Decis Mak.

